# Vitamin D Deficiency and Cardiovascular Mortality: Retrospective Analysis “Siena Osteoporosis” Cohort

**DOI:** 10.3390/nu15153303

**Published:** 2023-07-25

**Authors:** Filippo Pirrotta, Guido Cavati, Christian Mingiano, Daniela Merlotti, Ranuccio Nuti, Luigi Gennari, Alberto Palazzuoli

**Affiliations:** 1 Department of Medicine, Surgery and Neurosciences, University of Siena, 53100 Siena, Italy; pirrotta@student.unisi.it (F.P.); guido.cavati@student.unisi.it (G.C.); mingiano2@student.unisi.it (C.M.); daniela.merlotti@ao-siena.toscana.it (D.M.); ranuccio.nuti@unisi.it (R.N.); luigi.gennari@unisi.it (L.G.); 2 Cardiovascular Diseases Unit, Cardio-Thoracic and Vascular Department, Le Scotte Hospital, University of Siena, 53100 Siena, Italy

**Keywords:** cardiovascular diseases, vitamin D status, heart failure, mortality

## Abstract

Vitamin D is a fat-soluble vitamin that plays a key role in bone metabolism, particularly concerning the regulation of calcium and phosphate homeostasis. Cardiovascular disease (CVD) is the main cause of morbidity and mortality in Western countries. Knowledge of the role of vitamin D in CVD arose from evidence of the vitamin D receptor (VDR) inside the cardiovascular system. In this retrospective analysis, we investigated the relationships between vitamin D status and hospitalization for heart failure (HF), overall mortality and cardiovascular mortality. Between 2004 and 2009, age-stratified, random sampling of elderly men and postmenopausal women in the primary care registers of Siena residents was performed. In total, 174 males (mean ± SD, 65.9 ± 6 years) and 975 females (62.5 ± 6 years) were enrolled in the study. We investigated the association between 25OHD status and hospitalization for HF or causes of mortality. A total of 51 subjects (12 males and 39 females) had been hospitalized for acute HF. At the end of the survey, 931 individuals were alive, while 187 had died (43 males and 144 females). A greater proportion of deceased patients showed low 25OHD (particularly patients with levels below 20 ng/mL). A similar trend was observed concerning the prevalence of patients with 25OHD levels below 20 ng/mL who died from stroke (RR = 2.15; 95% CIs 0.98–4.69; *p* = 0.06). Low 25OHD levels may be predictive of cardiovascular mortality. Whether vitamin deficiency represents a primitive cause or is a simple bystander in increased cardiovascular mortality should be further investigated in prospective large cohort studies specifically designed to assess CVD risk, including a detailed assessment of cardiac dysfunction and the characterization of atherosclerotic lesions.

## 1. Introduction

Vitamin D is a fat-soluble vitamin that plays a key role in bone metabolism, particularly concerning the regulation of calcium and phosphate homeostasis [1]. Vitamin D can be obtained through the diet in the form of nutrients or it can be synthesized de novo in the human skin via exposure to ultraviolet B light (UVB) [2]. Dietary intake contributes to only 10–20% of human vitamin D supply; conversely, UVB-induced skin synthesis is the most important source of this prohormone. Ingested or light-acquired cholecalciferol is then transferred to the liver and bound to vitamin-D-binding protein. In the liver occurs the first round of hydroxylation with the transformation of cholecalciferol into 25 OH vitamin D (25OHD). In the kidney, 25OHD undergoes further hydroxylation into 1,25 dihydroxy-vitamin D (1,25OHD2), that is, the active form of vitamin D, which has an endocrine function in different target tissues [3].

Cardiovascular disease (CVD) is the main cause of morbidity and mortality in Western countries, despite improvements in current preventive and therapeutic strategies [4]. The increasing interest in the possible role of vitamin D in CVD arose from evidence of the vitamin D receptor (VDR) inside the cardiovascular system [4]. In particular, different reports confirmed the expression of VDRs in the human heart, myocardial tissue and various cells involved in cardiovascular functions [5,6]. Regarding these issues, there are two distinct aspects of this particular matter: one is the association of vitamin D deficiency with cardiovascular risk, and the other is the level of evidence concerning international studies on the usefulness of correcting vitamin D status in order to achieve a better cardiovascular outcome, which remains an open issue.

These observations led to the investigation of potential cardiovascular outcomes in the presence of inadequate vitamin D status, as defined by low serum levels of 25OHD, with conflicting results [7]. Indeed, a general consensus about optimal vitamin D levels has not been reached worldwide. While the Endocrine Society and other medical societies indicate 25OHD levels below 20 ng/mL as an indicator of vitamin D deficiency [8,9], the Institute of Medicine indicates a lower threshold of 12 ng/mL to define vitamin D deficiency [10].

However, irrespective of the given definition, poor vitamin D status is a common condition in the elderly population, affecting up to 50% of individuals in particular ethnicities and geographical areas when using the threshold of 20 ng/mL [11]. The reasons for this deficit are varied and include poor UVB exposure, inadequate dietary vitamin D intake, impaired vitamin D absorption, genetic abnormalities in hydroxylation and increased 25OHD catabolism [12]. Hypovitaminosis D and cardiovascular disease are both conditions with an increased prevalence, particularly in the elderly. Over the years, several studies have focused their attention on the possible correlation between low levels of vitamin D, increased cardiovascular risk beyond increased mortality for cardiovascular diseases (CVD) and all-cause mortality. Nevertheless, longitudinal studies do not uniformly agree on the recognition of vitamin D deficiency as an independent risk factor for CVD and heart failure incidence.

## 2. Aim of the Study

In this retrospective analysis, we investigated the relationships between vitamin D status and its impact on heart failure hospitalization, overall mortality and cardiovascular mortality using a large and well-characterized population-based cohort of elderly individuals.

## 3. Materials and Methods

### 3.1. Study Population

This is a retrospective study using longitudinal information derived from “Siena Osteoporosis” (SiOP). This is an ongoing study (started in 2003), performed with the collaboration of general practitioners from the Tuscany region, who randomly selected 1149 elderly men and postmenopausal women in their database, resulting in a representative sample for the evaluation of the prevalence of osteoporosis and bone fragility. Between 2004 and 2009, the collection of data was performed. The subjects of our cohort (between the ages of 50 and 80 years) were invited to undergo skeletal health assessment using a questionnaire, bone densitometry and blood tests for biochemical measurements, including 25OHD [13]. The study was conducted in accordance with the Declaration of Helsinki; ethics approval for the study was obtained in accordance with local institutional requirements (Prot. 961.03, first obtained on 29 October 2013), and written informed consent for inclusion was obtained from all participants.

At recruitment, height (measured using a stadiometer) and weight were recorded from all subjects. Information regarding sociodemographic and general health status, lifestyle, medical conditions and medications was collected using specific questionnaires. Dietary calcium intake was assessed using a validated food-frequency questionnaire including foods that account for the majority of calcium in the Italian diet [14]. In particular, information related to cardiovascular risk factors was also available (e.g., alcohol consumption, smoking, coffee consumption). Hypercholesterolemia, diabetes mellitus, hypertension, stroke history, atrial fibrillation, myocardial infarction and heart failure were also considered in the analysis. In total, 174 males (mean ± SD, 65.9 ± 6 years) and 975 females (62.5 ± 6 years) with available information were enrolled in the study.

In this study, we investigated the association between 25OHD status and hospitalization for heart failure or causes of mortality, starting from the beginning of the study at the baseline visit. Annually, verification of hospital admissions and dates of death were performed from the first year of recruitment (2004), until the last checkup date, on December 2022.

Patients with heart failure hospitalization were considered to include those who had logged into the Emergency Care Unit of Siena Hospital at any point during the entire observation time, presenting clinical heart failure symptoms and signs such as dyspnea, respiratory rales, hepato-jugular reflux, peripheral edema and orthopnea at the first clinical observation. We also collected a chest X-ray and biochemical blood tests executed in the Emergency Care Unit to confirm diagnosis. Another criterion for inclusion in this group was an ICD-9 diagnosis of heart failure at discharge. As reported in Table 1, a total of 51 subjects (12 males and 39 females) had been hospitalized for acute and symptomatic heart failure. Information about death and causes of mortality was obtained through ICD-9 codes related to hospital discharge forms and eventually confirmed by contacting the primary care physicians. Overall, as shown in Table 2, at the end of the survey, 931 individuals were alive (119 males and 812 females), while 187 had died (43 males and 144 females).

### 3.2. Biochemical Analyses

Serum alkaline phosphatase, aspartate and alanine aminotransferases, ionized and total calcium (colorimetric method, Roche, Auto-Analyzer Cobas 311, Basel, Switzerland), phosphate, and creatinine levels were determined using standard methods. Vitamin D status was assessed by measuring total 25OHD levels using ELISA methodology (DiaSorin Diagnostics, Saluggia, Italy; sensitivity, 1.5 ng/mL; interassay CV < 11%, intra-assay CV < 12.5%). The quality and accuracy of the 25OHD analyses from our laboratory are validated on an ongoing basis via participation in the External Quality Evaluation Program from the “Centro di Riferimento per la Qualità dei Servizi di Medicina di Laboratorio” [15]. According to the Endocrine Society (https://www.endocrine.org/ (accessed on 30 June 2023)) thresholds defining vitamin D status [8], we grouped individuals from the study cohort into the following 4 groups: group 1, including subjects with 25OHD values below 10 ng/mL; group 2, including subjects with 25OHD values between 10 and 20 ng/mL; group 3, including subjects with 25OHD values between 20 and 30 ng/mL; and group 4, including subjects with 25OHD values above 30 ng/mL.

### 3.3. Statistical Analysis

All analyses were performed using Statistica 10 (Statsoft, Tulsa, OK, USA) and SPSS (SPSS, version 21.0, IBM Corp., Armonk, NY, USA). Data were summarized as means ± standard deviations (SDs), and *p* < 0.05 was accepted as the value of significance. Quantitative variables were compared between the hospitalized and/or deceased individuals and control groups using analysis of variance and covariance, adjusting for age, BMI and lifestyle characteristics. Qualitative variables were compared using a chi-squared test. Logistic regression analysis was used to assess the independent association between vitamin D status (according to the 4 categories described above) and hospitalization for heart failure or mortality cause and presented as relative risk (RR) and 95% confidence intervals (CIs).

## 4. Results

Overall, 553 subjects in the study cohort (49.5%) had baseline 25OHD levels below 20 ng/mL, while 171 (15.3%) had severe vitamin D deficiency, with 25OHD levels below 10 ng/mL. During the longitudinal observation, 51 subjects reported hospitalization due to heart failure and 187 died. As shown in Table 1, the heart failure group presented similar general characteristics to the rest of the individuals from the Siena Osteoporosis cohort, apart from a slightly lower prevalence of obesity (12.9% vs. 5%) and higher prevalence of hypercholesterolemia (30.6% vs. 39.2%), diabetes mellitus (4.5% vs. 11.8%) and hypertension (17.5% vs. 23.5%) in the heart failure group.

Moreover, no significant differences were observed concerning serum calcium (9.27 ± 0.58 vs. 9.34 ± 0.66 mg/dL. *p* = 0.40), phosphate (3.42 ± 0.55 vs. 3.41 ± 0.59 mg/dL, *p* = 0.95) and alkaline phosphatase levels (180.57 ± 85.07 vs. 181.94 ± 64.8. *p* = 0.91) in patients with or without heart failure.

Conversely, no statistically significant differences in general characteristics or biochemical parameters were observed between deceased and living subjects. Both the populations presented no significant differences in serum calcium levels (9.29 mg/dL ± 0.59 vs. 9.25 mg/dL ± 0.56. *p* = 0.41) and alkaline phosphatase (178.63 Ui/L ± 86.29 vs. 187.23 ± 57.76 *p* = 0.18). Obesity was slightly higher in deceased subjects compared to living ones (10.8% vs. 13.4%); similar results were obtained for smoking (16.6% vs. 12.1%) and hypertension (15.2% vs. 17.1%). All the general characteristics of both populations are presented in Table 2.

**Figure 1:**
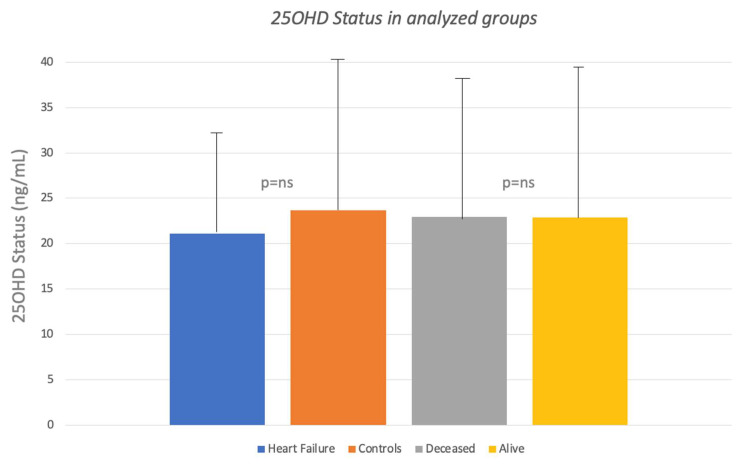
25OHD status in different groups analyzed. Heart failure patients present lower levels of vitamin D than controls, without significance. No significant differences were found between living and deceased groups.

**Figure 2:**
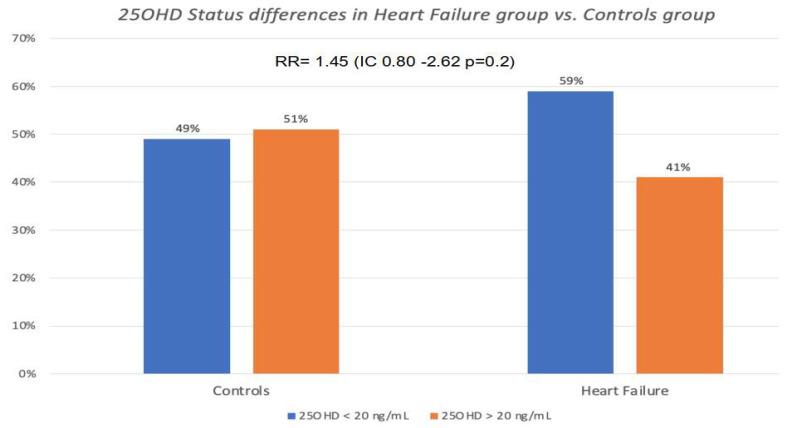
Levels of 25OHD levels between control group and heart failure group. In the control group, no particular differences were found in vitamin D levels. Patients hospitalized for heart failure presented 25OHD levels below 20 ng/mL compared to non-hospitalized patients, but without statistical significance (*p* = 0.2).

As shown in Figure 1, serum 25OHD levels assessed at the baseline visit were slightly but not significantly lower in the heart failure group and in deceased subjects than in the rest of the Siena Osteoporosis cohort (21.08 ng/mL SD ± 12.3.vs. 23.65 SD ± 17.5 ng/mL *p* = 0.2). As evident in Figure 2, 59.1% of heart failure cases had 25OHD levels below 20 ng/mL compared to 49.5% of subjects without hospitalization for heart failure (RR = 1.45; 95% CIs 0.80–2.62; *p* = 0.2).

We then evaluated the relationship between vitamin D status and mortality. In our cohort, among the 187 deceased patients, 50 (27%) died of cardiovascular disease, 28 died of stroke (15%), 38 (20%) died of cancer, 11 (6%) died of respiratory disease, and 60 (32%) died of other causes.

**Figure 3:**
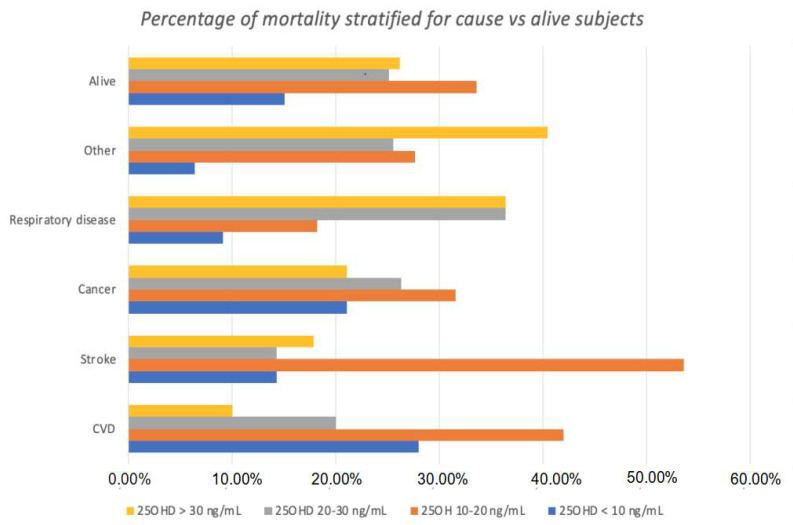
Number of events stratified by vitamin D status. Deceased subjects presented lower vitamin D levels compared to living subjects. Patients who were deceased due to cardiovascular diseases, including stroke, presented lower 25OHD levels than subjects who were deceased due to all the other causes. Most of these subjects were representative of group 3 and 4, with vitamin D levels below 20 ng/mL.

**Figure 4:**
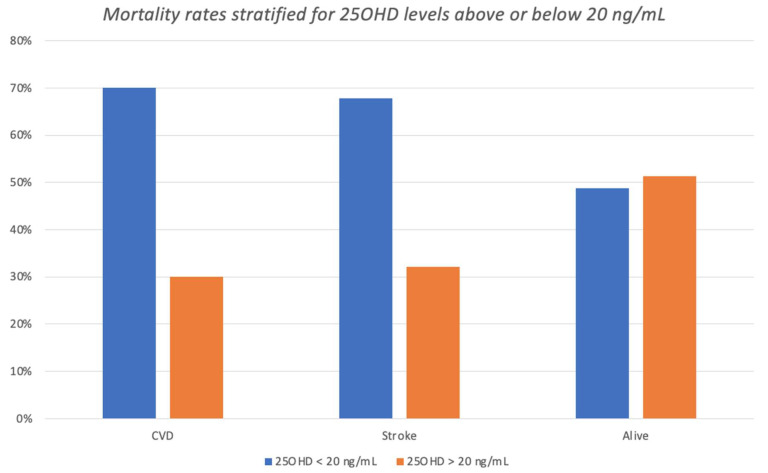
Number of events stratified by vitamin D status above or below 20 ng/mL. Patients who were deceased due to CVD and stroke were both representative of group 3 and 4 of vitamin D levels. Levels of vitamin D in living individuals were homogeneous.

Overall, 16.4% and 51.9% of the deceased subjects had 25OHD levels below 10 ng/mL or 20 ng/mL, respectively, compared with 15.1% and 48.9% of the living individuals. Figure 3 shows the differences in categories of 25OHD status the between deceased and living individuals, in relation to the main causes of mortality. Of interest, a greater proportion of deceased subjects showed 25OHD levels in the lower groups (group 1: 25OHD < 10 ng/mL and group 2: 25OHD ≥ 10 and <20 ng/mL). This was equivalent to an RR of 2.32 (95% CIs 1.28–4.18; *p* = 0.005), with a number needed to harm of 21.5 (95% CIs 66.0–12.8) when considering all cases with 25OHD below 20 ng/mL (groups 1 and 2), as shown in Figure 4. A similar trend was observed concerning the prevalence of patients with 25OHD levels below 20 ng/mL who died of stroke (RR = 2.15; 95% CIs 0.98–4.69; *p* = 0.06).

## 5. Discussion

During the last two decades, the role of vitamin D deficiency in cardiovascular disease has been widely debated, and although different studies have reported a close association between vitamin D deficiency and increased cardiovascular risk, the relationship remains controversial. Since both heart failure (HF) and osteoporosis are common disorders in the elderly, the role of vitamin D deficiency in this field remains questioned, and the presence of low 25OHD levels in patients with CVD might indeed represent the consequence rather than the cause of the disease. The results of our study on a representative population-based cohort did not find a statistically significant correlation of the presence of low 25OHD levels with a greater incidence of hospitalization due to heart failure, nor with overall mortality. However, we demonstrated that low 25OHD levels (below 20 ng/mL) might be predictive of increased cardiovascular mortality due to heart disease and possibly stroke. Taken together, these results are consistent with a larger analysis on the prospective cohort study of 307,601 individuals from the UK Biobank, showing a statistically significant association between vitamin D status and mortality, including cardiovascular mortality [16]. Indeed, in the same cohort, similar conclusions were reached when genetically predicted 25OHD was calculated using a non-linear mendelian randomization approach, thus supporting a causal relationship between vitamin D deficiency and mortality. Of interest, in the same cohort, vitamin D status also predicted the risk of recurrent cardiovascular events, with a potential threshold around 20 ng/mL [17]. Indeed, both the UK and Italian populations are considered at particularly higher risk of vitamin D deficiency, particularly during the winter season, when the skin is unable to produce adequate vitamin D levels, and since dietary vitamin D intake is limited due to the lack of adequate food fortification.

The potential mechanisms involved in the onset and evolution of CVD mediated by vitamin D deficiency are varied. VDRs seem to play a role in the regulation of renin–angiotensin–aldosterone (RAAS) and, hence, in arterial pressure control. Studies conducted that focused on hypertension in mice showed the development of high blood pressure and cardiac hypertrophy linked to increased RAAS activation in VDR knock-out mice [18]. Other studies evidenced vitamin D’s role in the induction of angiotensin-converting enzyme 2, which is responsible for the cleavage of angiotensin II to angiotensin 1–7, [19] a metabolite involved in anti-hypertensive effects such as natriuresis, vasodilation and reduced free oxygen radical species. According to our findings, an observational study conducted by Wang et al. [20] attributed vitamin D deficiency to an increased prevalence of hypertension and RAAS activation, leading to a rise in cardiovascular events and mortality. Another effect appears linked to endothelial dysfunction, driven by the reduction in nitric oxide synthase (eNOS) production with consequent vascular tone increase and stress [21].

Moreover, atherosclerosis is mostly related to vascular damage, which involves increased plaque formation and has a significant impact on cardiac and cerebrovascular events such as stroke and myocardial infarction. An important role in plaque rupture and instability is played by inflammation. The formation of atheroma is also related to macrophage-derived foam cell formation at the site of the damaged endothelium. Vitamin D was shown to reduce cholesterol accumulation in macrophages and LDL uptake in atheromas [22]. Additionally, 1,25OHD2 is involved in the reduced expression of matrix metalloproteinase (MMP)-2 and MMP-9 and in the improvement of inhibitor activation, thereby preventing plaque destabilization and luminal rupture [23]. The anti-inflammatory action of vitamin D is largely known: several in vitro studies demonstrate the direct action of vitamin D in the down-regulation of nuclear factor-κB activity, leading to a decrease in the formation of inflammatory cytokines, chemokines and molecular pathways (IL-6, IL-12, interferon-γ and TNF-α production). Moreover, the stimulation of anti-inflammatory products (IL-10) strengthens the association with reduced cytokine response [24,25,26,27]. Vitamin D can contribute to thrombomodulin and tissue factor expression regulation, leading to an anticoagulant effect and preventing thrombogenic activity, one of the factors most responsible for myocardial infarction [28]. Finally, excess PTH levels related to chronic vitamin D deficiency can represent an important factor in the development of CVD in humans, as well as cardiomyocyte hypertrophy, interstitial cardiac fibrosis, alterations in myocardial contractility, Ca^2+^ signal-related myocardial tone, vessel calcification and all other pro-arrhythmic effects related to increased sarcoplasmic calcium intake [29]. In the elderly, CVD and low vitamin D levels may frequently coexist. Patients with chronic heart failure have a reduction in terms of mobility, and this condition may result in lowered UV exposure, which, in turn, leads to muscle catabolism, sarcopenia and bone frailty [30]. Additionally, loss of muscle skeletal mass and strength lead to poor exercise capacity and reduced mobilization directly proportional to the severity of heart failure [31]. The link occurring between a poor vitamin D status and the occurrence of heart failure results from higher wall tension, increased fibrosis and vascular stiffness [32]. Therefore, the above-cited pro-atherosclerotic process is an additional factor in heart failure development. In this respect, our results show a trend towards an increase in heart failure events among subjects with 25OHD levels below 20 ng/mL, although this did not reach statistical significance, in part due to the limited number of cases with reported hospitalization due to acute heart failure.

Finally, hypovitaminosis D induces bone fragility and higher fracture risk that, in turn, lead to bed rest, prolonged immobilization and muscular tone deactivation. A study conducted by Carbone et al. evidenced an increase in mortality in patients with heart failure who sustained hip fractures, compared to patients without this event, and the results showed about a two-fold increase in mortality in the patients with fractures [33]. The current findings appear much more pronounced in white males and in subjects with low bone mineral density (BMD) [34]. Notably the role of BMD as a possible marker of heart failure-related events is contrasting. Jankowska et al. found a BMD value reduction associated with heart failure severity and sarcopenia [35]. Loncar et al. [36], in a small male sample size, confirmed the role of low BMD as a risk factor for increased mortality. They also found an independent association between BMD reduction and elevated systolic blood pressure, elevated arterial stiffness and LV diastolic dysfunction. Accordingly, Fohtung et al. in a homogeneous population, demonstrated that lower BMD values were independently associated with higher heart failure risk in non-black men, but a lower risk in black men [34].

Overall, our study has several limitations. First, the study was originally designed to be a screening of the risk of osteoporosis and fractures, and the collection of risk factors and cardiovascular events was performed indirectly using questionnaire collection and an analysis of ICD-9 information at hospital discharge. Specifically designed approaches should be required to better address the relationship between vitamin D levels and the morphological or functional cardiac alterations that underlie heart failure. In fact, since this is a retrospective analysis of an epidemiological cohort study, it was not able to discern between different heart failure subtypes and the main pathophysiological mechanism. Due to the observational nature of the study, only acute events requiring hospitalization were accounted for, whereas early heart failure stages and asymptomatic patients were not considered. We also underline the disparity in our population characterized by the prevalence of female sex. The presence of gender-related differences in the onset, symptoms and prevalence of cardiovascular diseases is well known in the literature [37]. For this reason, a future investigation should better address the relationship between sex and increasing CVD events according to different vitamin D levels. Finally, a single 25OHD measurement could not correctly reflect vitamin D status across the large observation period of our retrospective analysis, and it was not possible to consider the use of vitamin D supplementation.

## 6. Conclusions

In conclusion, notwithstanding the above limitations, the information derived from our retrospective analysis on a large and well-characterized cohort, for more than 10 years of observation, suggest that low 25OHD levels may be predictive of cardiovascular mortality. Whether vitamin D deficiency represents a primitive cause or is a simple bystander in increased cardiovascular mortality should be further investigated in prospective large cohort studies specifically designed to assess CVD risk, including a detailed assessment of cardiac dysfunction and the characterization of atherosclerotic lesions.

## Figures and Tables

**Table 1:** Main characteristics of control group vs. heart failure group.

*n*	Control Group	Heart Failure Group	*p* Value
Females	797 (83.9%)	39 (76.5%)	
Males	153 (16.1%)	12 (23.5%)	
Age at baseline (yrs)	63.14 ± 6.76	62.95 ± 7.12	
BMI (Kg/m^2^)	25.72 ± 4.2	25.9 ± 3.18	n.s.
Obesity (BMI > 30 Kg/m^2^)	123 (12.9%)	3 (5.9%)	
Smoke (%)	127 (13.4%)	6 (11.8%)	
Hypertension (%)	166 (17.5%)	12 (23.5%)	
Ipercholesterolemia (%)	291 (30.6%)	20 (39.2%)	
Diabetes (%)	43 (4.5%)	6 (11.8%)	
Chronic Kidney Disease (%)	12 (1.3%)	1 (2%)	
Calcium Intake (mg/day)	837.07 ± 319.43	836.40 ± 345.68	n.s.
Calcium (mg/dL)	9.27 ± 0.58	9.34 ± 0.66	n.s.
Phosphorus (mg/dL)	3.42 ± 0.55	3.41 ± 0.59	n.s.
Alkaline Phosphatase (UI/L)	180.57 ± 85.07	181.94 ± 64.84	n.s.
25OH Vitamin D (ng/mL)	23.65 ±16.5	21.08 ± 12.4	n.s.

n.s. = non significant.

**Table 2:** Main characteristics of the two groups analyzed: alive and deceased groups.

*n*	Alive Subjects	Deceased Subjects	*p* Value
Females (%)	812 (87.2%)	144 (77%)	
Males (%)	119 (12.3%)	43 (23%)	
Age at baseline (yrs)	62.21 ± 6.31	66.4 ± 6.41	<0.05
BMI (Kg/m^2^)	25.61 ± 4.17	25.81 ± 4.35	n.s
Obesity (BMI > 30 Kg/m^2^)	101 (10.8%)	25 (13.4%)	
Smoke (%)	113 (12.1%)	31 (16.6%)	
Hypertension (%)	151 (15.2%)	32 (17.1%)	
Ipercholesterolemia (%)	270 (29%)	56 (29.9%)	
Diabetes (%)	40 (4.3%)	10 (5.3%)	
Chronic Kidney Disease (%)	8 (0.9%)	5 (2.7%)	
Calcium Intake (mg/day)	842.52 ± 329.71	806.72 ± 276.07	n.s
Calcium (mg/dL)	9.29 ± 0.59	9.25 ± 0.56	n.s
Phosphorus (mg/dL)	3.44 ± 0.55	3.35 ± 0.54	
Alkaline Phosphatase (UI/L)	178.63 ± 86.29	187.23 ± 57.76	n.s
Creatinine (mg/dL)	0.91 ± 0.33	0.96 ± 0.21	<0.05
25OH Vitamin D (ng/mL)	22.89 ± 16.7	22.96 ± 14.8	n.s

n.s. = non significant.

## Data Availability

Not available due to patients’ privacy reasons.
